# Parent-adolescent relationship quality and smartphone addiction: a hierarchical analysis of relational and demographic predictors

**DOI:** 10.3389/fpubh.2026.1749726

**Published:** 2026-03-27

**Authors:** C. Anbumalar, D. Binu Sahayam

**Affiliations:** School of Social Sciences and Languages, Vellore Institute of Technology, Chennai, India

**Keywords:** adolescent smartphone addiction, family structure, gender differences, hierarchical regression, parent–adolescent relationship, parental support, paternal alienation

## Abstract

**Background:**

Adolescents’ smartphone use has increased substantially, raising concerns about problematic use and its public health implications. Although parent–child relationship quality plays an important role in adolescents’ behavioral regulation, the distinct contributions of parental support and alienation to smartphone addiction remain insufficiently clarified.

**Objective:**

This study examined whether maternal and paternal support and alienation were associated with adolescent smartphone addiction after accounting for demographic characteristics and smartphone use duration.

**Methods:**

A cross-sectional study was conducted among 210 adolescents (aged 11–16 years) from government and government-aided schools in Chennai, India. Participants completed the Smartphone Addiction Scale–Short Version–Tamil (SAS-SV-T) and the mother and father forms of the Inventory of Parent and Peer Attachment (IPPA). Descriptive statistics, Pearson correlations, hierarchical multiple regression analyses (maternal and paternal models), and binary logistic regression were performed using SPSS 25.0.

**Results:**

Male gender and longer duration of smartphone use were consistently associated with higher smartphone addiction severity and risk. In hierarchical models, relational variables explained additional variance beyond demographic factors. Paternal alienation emerged as a significant positive predictor of addiction severity, whereas maternal support demonstrated a modest protective association. Family structure did not independently predict smartphone addiction outcomes.

**Conclusion:**

Adolescent smartphone addiction is shaped not only by behavioral exposure and demographic factors but also by the emotional quality of parent–adolescent relationships. Perceived paternal alienation was associated with greater addiction severity. Prevention efforts may benefit from integrating relational-strengthening strategies alongside digital-behavior regulation.

## 1 Introduction

Adolescence is a period of rapid physiological, psychological, and social transitions and maturation (1). During this developmental period, young people increasingly seek autonomy while continuing to rely on caregivers for emotional support and regulation. This period is also marked by increased vulnerability to digital engagement, especially smartphone use, which has become a widely used medium for communication, entertainment, and academic work (2, 3).

Smartphone addiction is conceptualized as a pattern of compulsive use characterized by functional impairment and difficulty controlling usage. It can arise from overuse or dysregulated use of smartphones, despite their educational and social benefits (4–7). Global evidence shows that problematic smartphone use among adolescents is becoming more common (8), with higher risks seen in Asian contexts where digital access is growing quickly (9, 10). Therefore, understanding adolescent smartphone addiction requires attention not only to behavioral exposure but also to relational and developmental contexts.

The quality of the parent–child relationship is considered a key protective or risk factor in shaping adolescent behavioral outcomes. Secure attachment, resilience, and adaptive emotional regulation are all influenced by parental emotional availability, communication, warmth, and responsiveness (11). On the other hand, adolescents who experience parental alienation, which includes hostility, emotional withdrawal, or psychological unavailability, are more vulnerable to stress, internalizing symptoms, and maladaptive coping strategies (12, 13). Studies show that adolescents who have supportive relationships with their parents have lower levels of digital dependence, while those who have conflict, rejection, or alienation are more prone to excessive smartphone use (14–16).

Theoretical viewpoints further explain these relational mechanisms. Attachment theory posits that emotionally responsive caregiving fosters internal regulatory capacity, whereas relational disconnection may heighten vulnerability to maladaptive coping strategies (17, 18). Within digital contexts, unmet attachment needs may increase reliance on online environments for temporary regulation or validation. The Compensatory Internet-Use Theory (CIUT) similarly proposes that individuals may engage in excessive online behavior as a coping response to interpersonal distress or unmet offline needs (19). These frameworks explain how parental alienation may increase adolescents’ dependence on smartphones for a sense of support, escape, and validation, while parental support may act as a protective relational resource (20, 21).

Although maternal support has received comparatively greater empirical attention, emerging evidence highlights the distinct role of fathers in adolescent development. Paternal involvement has been associated with social competence, behavioral adjustment, and emotion regulation (22, 23). Studies across diverse cultural settings indicate that father-adolescent alienation may be linked to increased vulnerability to smartphone addiction (15, 16). However, paternal relational dynamics remain underexamined in many non-Western contexts.

Family structure has been examined as a contextual factor in adolescent outcomes. While some studies suggest that single-parent households may experience reduced supervisory or emotional resources, accumulating evidence indicates that relational quality within the family is more predictive of adolescent adjustment than structural configuration alone (15, 24). Accordingly, family structure may operate as a contextual condition shaping relational processes rather than functioning as a direct determinant of behavioral risk.

Addiction risk is also influenced by smartphone usage patterns and gender. Though the research findings are mixed, many studies report higher addiction levels among male adolescents (25, 26). Other studies report no gender differences or higher risk among females (27, 28). Owning a smartphone for a longer period increases the likelihood of problematic use (29). Accordingly, demographic and usage-related factors must be considered when examining relational predictors of smartphone addiction.

Adolescence is a developmental stage in which youth continue to rely on caregivers for emotional reassurance and regulation, even as they strive for greater independence. In families where two caregivers are present, difficulties in one parent–child relationship may be softened by the emotional availability of the other parent, allowing adolescents to maintain a sense of relational balance (22, 23). However, in single-parent households, particularly when fathers are non-resident or emotionally distant, such buffering opportunities may be reduced (24, 30). As a result, experiences of paternal alienation may carry greater emotional weight because adolescents have fewer alternative attachment figures within the family system. The Compensatory Internet-Use Theory suggests that when interpersonal needs are not adequately met offline, individuals may turn to digital environments for comfort, validation, or distraction (19). Empirical findings further indicate that insecure parental attachment is associated with problematic smartphone use through maladaptive emotion regulation processes (15, 20). Together, these perspectives suggest that family structure may not directly determine addiction risk; rather, it may shape how strongly relational experiences, especially paternal alienation, affect adolescents’ tendency to rely on smartphones as an emotional coping tool.

The present study examined how maternal and paternal relationship quality, operationalized as parental support (trust and communication) and parental alienation, relates to adolescent smartphone addiction. Drawing on attachment theory and Compensatory Internet-Use Theory, we focused on two central relational dimensions: parental support (emotional closeness, trust, and communication) and parental alienation (feelings of emotional distance or disconnection). These frameworks suggest that when adolescents experience unmet attachment needs or emotional strain within the family, they may turn to digital environments as alternative sources of comfort, validation, or escape.

In addition to relational variables, demographic and contextual influences were examined. Gender differences and duration of smartphone use were considered established risk factors. Family structure was included not as a direct determinant of addiction, but as a contextual variable within which relational dynamics unfold. Consistent with attachment-based perspectives, this study emphasizes that emotional quality within parent–adolescent relationships may be more consequential than structural family configuration.

To our knowledge, few studies in the Indian context have simultaneously examined maternal and paternal relational dimensions using hierarchical modeling to differentiate relational effects from demographic exposure variables.

Accordingly, the study tested the following hypotheses:

*H1*: Higher levels of parental support (maternal and paternal) would be associated with lower levels of smartphone addiction.

*H2*: Higher levels of parental alienation (maternal and paternal) would be associated with higher levels of smartphone addiction.

*H3*: Male gender and longer duration of smartphone use would be associated with higher levels of smartphone addiction.

## 2 Materials and methods

### 2.1 Study design

This study employed a cross-sectional, school-based analytical survey design to examine the association between parent–adolescent relationship quality and adolescent smartphone addiction.

### 2.2 Participants

Participants were recruited from six government and government-aided schools across multiple educational districts in Chennai, India. Prior to data collection, administrative approval was obtained from the Chief Educational Officer (CEO) of the Chennai district. Schools were organized geographically into North, South, and Central divisions to enhance regional representation. Within each division, schools that granted institutional permission were included.

Students enrolled in grades 7 to 9 were invited to participate.

Inclusion criteria

- Enrollment in a government or government-aided school in Chennai
- Studying in grades 7–9
- Living with at least one biological parent

Exclusion criteria

- Adolescents residing primarily with non-parental guardians
- Students with diagnosed developmental or neurological conditions, as reported by school authorities

A total of 210 adolescents participated. The mean age was 13.25 years (*SD* = 0.96; range = 11–16). Of the sample, 114 (54.3%) were female, and 96 (45.7%) were male. Family structure was evenly distributed, with 105 adolescents (50%) from single-parent households and 105 (50%) from dual-parent households.

### 2.3 Measures

#### 2.3.1 Sociodemographic variables

Participants provided basic information, including age, gender, class, family structure (single or dual parent), daily hours of smartphone use, years of smartphone use, and primary purpose of smartphone use. Years of smartphone use were treated as a continuous predictor variable in regression analyses.

#### 2.3.2 Parent–adolescent relationship quality

Parent–adolescent relationship quality was assessed using the mother and father forms of the Inventory of Parent and Peer Attachment–Revised [IPPA-R; (31, 32)]. Each form consists of 25 items rated on a five-point Likert scale and assesses three dimensions: trust, communication, and alienation.

- Trust reflects perceived reliability and emotional security (e.g., “My parent respects my feelings”).
- Communication reflects openness and quality of dialogue (e.g., “I can talk to my parent about my problems”).
- Alienation reflects feelings of anger, emotional distance, or disconnection (e.g., “I feel angry with my parent”).

Consistent with attachment theory, the trust and communication subscales were summed to create composite indices of maternal and paternal support. These dimensions collectively reflect emotional availability and relational security, core components of attachment-based support (17, 18). Composite scores were computed by summing the items on the trust and communication subscales to form maternal and paternal support indices. Internal consistency for the composite support indices was satisfactory. The alienation subscale was retained as a separate construct for both mother and father.

The IPPA-R mother and father forms were translated into Tamil using forward–back translation procedures by bilingual psychologists. The translated version underwent expert review and pilot testing to ensure cultural appropriateness and clarity for Tamil-speaking adolescents.

Combining the Trust and Communication subscales into a composite parental support index is supported by cross-cultural validation studies demonstrating a two-factor structure (Support vs. Alienation) with strong model fit and high intercorrelations between trust and communication dimensions (33, 34). These findings are consistent with attachment theory, which conceptualizes emotional availability and relational security as unified components of supportive caregiving (17, 18). Accordingly, trust and communication were summed to form maternal and paternal support indices, while alienation was retained as a distinct construct.

#### 2.3.3 Internal consistency

In the present sample:

- Maternal support demonstrated good internal consistency (*α* ≈ 0.84)
- Paternal support demonstrated high internal consistency (*α* ≈ 0.90)
- Maternal alienation demonstrated acceptable internal consistency (*α* ≈ 0.73).
- Paternal alienation demonstrated acceptable internal consistency (*α* ≈ 0.74).

#### 2.3.4 Smartphone addiction

Adolescent smartphone addiction was measured using the Smartphone Addiction Scale–Short Version (SAS-SV) (7). The 10-item instrument assesses behavioral dependence and difficulty in controlling smartphone use, with responses rated on a 6-point Likert scale from 1 (“strongly disagree”) to 6 (“strongly agree”). The Tamil version of the Smartphone Addiction Scale -Short Version (SAS-SV-T) developed by Anbumalar and Binu Sahayam was used for this study (35). Internal consistency was satisfactory in this study (Cronbach’s *α* = 0.744). Gender-specific cut-off scores were applied to classify adolescents into addiction risk categories.

### 2.4 Procedure

Data were collected during school hours in classroom settings. Students were given the questionnaire that included smartphone addiction and mother and father attachment, which could be completed in approximately 15 min. All students received the same test and instructions. The survey was administered in Tamil, and the researcher was present to clarify any procedural questions.

### 2.5 Data analysis

Data were analyzed using SPSS version 25.

- Descriptive statistics were computed for all study variables.
- For non-resident parents, parental attachment variables were coded as system-missing to ensure analyses reflected only available relational data.
- Pearson correlation analyses were conducted to examine bivariate associations. Due to differential availability of parental data (e.g., absence of one parent in some households), Pearson correlations were computed using pairwise deletion to maximize available information across variables. Hierarchical multiple regression analyses were performed separately for maternal (*N* = 187) and paternal (*N* = 128) models due to differential availability of parental data. Separate maternal and paternal models were estimated to avoid unnecessary reduction of sample size through listwise deletion and to examine parent-specific relational dynamics independently. Patterns of associations were consistent across available subsamples, reducing concern that differential parental data availability introduced systematic bias in model estimation.
- In each regression model, demographic variables (age, gender, years of smartphone use, family structure) were entered in Block 1, followed by relational variables in Block 2.
- Multicollinearity was assessed using variance inflation factors (VIF).
- Binary logistic regression was conducted to examine predictors of smartphone addiction risk classification.
- Statistical significance was set at *p* < 0.05.

### 2.6 Ethical considerations

The study was conducted in accordance with the Declaration of Helsinki and the Indian Council of Medical Research. The Institutional Ethical Committee for Research in Human Subjects of the Vellore Institute of Technology, Chennai Code, VIT/IECH/CC/2024/95, approved the study. The research was conducted in accordance with the local legislation and institutional requirements. Written informed consent was obtained from the school principal and parents, and assent was obtained from all adolescent participants.

## 3 Results

### 3.1 Participant characteristics

The demographic characteristics of the sample are presented in Table 1. The study included 210 adolescents (54.3% female; 45.7% male) with a mean age of 13.25 years (*SD* = 0.96). The average duration of smartphone use was 4.65 years (*SD* = 2.60). Approximately half of the participants (50%) belonged to single-parent households and 50% to dual-parent households. Nearly half of the adolescents (48.6%) were classified as being at risk for smartphone addiction based on gender-specific cut-off scores.

**Table 1:** Demographic characteristics of the participants (*N* = 210).

Variable	Category	*n*	%	Mean (*SD*)
Age (years)	—	—	—	13.25 (0.96)
	11	7	3.3	
	12	37	17.6	
	13	80	38.1	
	14	71	33.8	
	15	13	6.2	
	16	2	1	
Gender	Male	96	45.7	
	Female	114	54.3	
Grade	7th	54	25.7	
	8th	74	35.2	
	9th	82	39	
Daily smartphone use	≤1 h	131	62.4	
	2–3 h	52	24.8	
	≥4 h	27	12.8	
Years of smartphone use	—	—	—	4.65 (2.60)
Purpose of smartphone use	Education only	20	9.5	
	Games only	44	21	
	Entertainment only	33	15.7	
	Multiple purposes	113	53.8	
Family structure	Single- parent	105	50	
	Dual- parent	105	50	
Addiction risk	No risk	108	51.4	
	At risk	102	48.6	

*N* = 210 adolescents. Percentages are calculated within each variable. Mean and standard deviation (*SD*) are reported for continuous variables (age and years of smartphone use). Daily smartphone use refers to average time spent per day. Addiction risk classification is based on the cut-off criteria of the standardized smartphone addiction scale used in the study.

### 3.2 Correlational findings

Pearson correlations among study variables are presented in Table 2. Years of smartphone use was positively associated with smartphone addiction (*r* = 0.29, *p* < 0.01). Mother support was negatively associated with smartphone addiction (*r* = −0.19, *p* < 0.01). Father alienation was positively associated with smartphone addiction (*r* = 0.26, *p* < 0.01), indicating that higher perceived paternal alienation was associated with greater smartphone addiction severity.

**Table 2:** Pearson correlations among study variables.

S. No	Variable	*N*	*M*	*SD*	1	2	3	4	5	6	7
1.	Age	210	13.25	0.96	—						
2.	Years of Smartphone use	210	4.65	2.60	−0.03	—					
3.	Smartphone addiction	210	30.72	9.76	0.10	0.29**	—				
4.	Mother support	187	72.94	12.87	0.11	−0.14	−0.19**	—			
5.	Father support	128	69.89	15.38	−0.07	−0.04	−0.07	0.27**	—		
6.	Mother alienation	187	18.25	5.83	0.09	−0.06	0.07	−0.05	−0.02	—	
7.	Father alienation	128	18.46	5.98	0.06	0.08	0.26**	−0.09	0.24**	0.31**	—

Ns ranged from 128 to 210 due to the differential availability of parental data. Correlations were computed using pairwise deletion. Values represent Pearson’s r. Two-tailed tests. **p < 0.01.

Father support was negatively associated with father alienation (*r* = −0.24, *p* < 0.01), consistent with the conceptual distinction between supportive and alienating parental behaviors. Mother–father alienation was moderately positively correlated (*r* = 0.31, *p* < 0.01).

Correlations involving parental variables varied in sample size because adolescents from single-parent households reported data only for the resident parent. Effect sizes were small to moderate in magnitude (Table 2).

### 3.3 Hierarchical multiple regression

To examine whether parent–adolescent relationship variables predicted smartphone addiction after controlling for demographic characteristics, hierarchical multiple regression analyses were conducted separately for maternal and paternal models, given differences in available parental data.

#### 3.3.1 Maternal model (*N* = 187)

In Model 1, age, gender, years of smartphone use, and family structure explained 21.9% of the variance in smartphone addiction, *F* (4,182) = 12.78, *p* < 0.001. Male gender (*β* = 0.29, *p* < 0.001) and years of smartphone use (*β* = 0.26, *p* < 0.001) were significant predictors, whereas age was not significant. Family structure showed a small negative effect in Model 1 (*β* = −0.14, *p* = 0.039) (Table 3).

**Table 3:** Hierarchical regression predicting smartphone addiction (maternal model, N = 187).

Predictor	*B*	*SE B*	*β*	*t*	*p*
Model 1: Demographics					
Age	0.95	0.66	0.10	1.43	0.153
Gender (1 = Male)	5.48	1.29	0.29	4.26	< 0.001
Years of smartphone use	0.95	0.25	0.26	3.78	< 0.001
Family structure (1 = Single-parent)	−2.67	1.29	−0.14	−2.08	0.039
Model 2: Maternal variables					
Mother support	−0.11	0.05	−0.14	−2.13	0.035
Mother alienation	0.16	0.11	0.10	1.53	0.127

Model fit: Model 1: R^2^ = 0.219, F (4,182) = 12.78, *p* < 0.001; Model 2: R^2^ = 0.250, ΔR^2^ = 0.031, *p* = 0.027.

In Model 2, maternal support and maternal alienation were added. The addition of maternal relational variables significantly improved the model (*ΔR^2^* = 0.03, *p* = 0.027), with the full model explaining 25.0% of the variance. Maternal support emerged as a significant negative predictor of smartphone addiction (*β* = −0.14, *p* = 0.035), whereas maternal alienation was not significant. Multicollinearity diagnostics indicated no concerns, with VIF values ranging from 1.02 to 1.10. Family structure was no longer significant after relational variables were included.

#### 3.3.2 Paternal model (*N* = 128)

In Model 1, age, gender, years of smartphone use, and family structure explained 22.1% of the variance in smartphone addiction*, F* (4,123) = 8.72, *p* < 0.001. Age (*β* = 0.22, *p* = 0.011), male gender (*β* = 0.22, *p* = 0.008), and years of smartphone use (*β* = 0.31, *p* < 0.001) were significant predictors. Family structure was not significant.

In Model 2, paternal support and paternal alienation were entered. The addition of paternal relational variables significantly increased explained variance (*ΔR^2^* = 0.055, *p* = 0.012), with the full model accounting for 27.6% of the variance in smartphone addiction.

Paternal alienation emerged as a significant positive predictor (*β* = 0.24, *p* = 0.003), indicating that higher perceived alienation from fathers was associated with greater smartphone addiction severity. Paternal support was not significant in the final model. In the paternal model, VIF values ranged from 1.08 to 1.19, indicating acceptable tolerance levels and no evidence of multicollinearity. Paternal alienation remained a significant predictor after controlling for demographic variables (Table 4).

**Table 4:** Hierarchical regression predicting smartphone addiction (paternal model, N = 128).

Predictor	*B*	*SE B*	*β*	*t*	*p*
Model 1: Demographics					
Age	2.29	0.89	0.22	2.57	0.011
Gender (1 = Male)	4.59	1.70	0.22	2.70	0.008
Years of smartphone use	1.17	0.32	0.31	3.67	<0.001
Family structure (1 = Single-parent)	1.99	2.21	0.08	0.90	0.368
Model 2: Paternal variables					
Father support	−0.07	0.05	−0.10	−1.22	0.224
Father alienation	0.42	0.14	0.24	3.01	0.003

Model Fit: Model 1: R^2^ = 0.221, F (4,123) = 8.72, *p* < 0.001; Model 2: R^2^ = 0.276, ΔR^2^ = 0.055, *p* = 0.012.

Higher alienation reflects greater perceived emotional distance.

B = unstandardized coefficient; SE B = standard error; β = standardized coefficient. Gender was coded 1 = male and 0 = female. Family structure was coded 1 = single-parent and 0 = dual-parent households.

Family structure did not significantly predict smartphone addiction in either maternal or paternal models after relational variables were entered, and no interaction effects were tested.

### 3.4 Binary logistic regression predicting addiction risk

Binary logistic regression was conducted to examine predictors of smartphone addiction risk classification. The overall model was significant, *χ^2^*(6) = 36.84, *p* < 0.001. The model explained between 25.0% (Cox & Snell *R^2^*) and 33.5% (Nagelkerke *R^2^*) of the variance and demonstrated adequate fit (Hosmer–Lemeshow *p* = 0.369).

Male adolescents were significantly more likely to be classified as at risk compared to females (*OR* = 3.10, *p* = 0.009). Age (*OR* = 1.80, *p* = 0.012) and years of smartphone use (*OR* = 1.36, *p* = 0.001) significantly increased the likelihood of addiction risk.

Paternal alienation approached significance (*OR* = 1.07, *p* = 0.072), but parental relational variables and family structure were not significant predictors of categorical addiction risk (Table 5).

**Table 5:** Binary logistic regression predicting smartphone addiction risk.

Predictor	*B*	*SE*	*OR*	95% *CI*	*p*
Age	0.59	0.23	1.80	1.14–2.83	0.012
Gender (1 = Male)	1.13	0.43	3.10	1.33–7.21	0.009
Years of smartphone use	0.31	0.09	1.36	1.14–1.62	0.001
Family structure (1 = Single- parent)	−0.01	0.57	0.99	0.33–3.00	0.987
Father support	0.00	0.01	1.00	0.98–1.03	0.883
Father alienation	0.07	0.04	1.07	0.99–1.15	0.072

Model Fit: χ^2^(6) = 36.84, *p* < 0.001; Nagelkerke R^2^ = 0.335; Hosmer–Lemeshow *p* = 0.369.

Addiction risk was classified using gender-specific SAS-SV cut-off scores.

OR = odds ratio; CI = confidence interval. Gender was coded 1 = male and 0 = female. Family structure was coded 1 = single-parent and 0 = dual-parent households. Smartphone addiction risk was classified using gender-specific cut-off scores on the Smartphone Addiction Scale–Short Version (≥31 for males; ≥33 for females). Nagelkerke R^2^ = 0.335. The Hosmer–Lemeshow goodness-of-fit test was non-significant (*p* = 0.369), indicating adequate model fit.

## 4 Discussion

This study examined adolescent smartphone addiction within the emotional context of parent–adolescent relationships, extending models that focus primarily on screen exposure. After accounting for gender, age, and duration of smartphone use, paternal alienation and maternal support showed distinct associations with addiction severity, whereas family structure did not independently predict outcomes. These patterns highlight the importance of relational dynamics over structural configuration in understanding adolescents’ digital behavior.

### 4.1 Sociodemographic trends and patterns of smartphone use

The present sample comprised early to mid-adolescents (*M* age = 13.25 years), a developmental period characterized by heightened sensitivity to peer influence, increasing autonomy, and ongoing maturation of executive control systems. From a developmental psychopathology perspective, this stage represents a window of both opportunity and vulnerability. The integration of smartphones into daily routines during this period is therefore not incidental but developmentally embedded.

Most adolescents reported daily smartphone use of at least 1 hour, with an average ownership history approaching 5 years. This indicates exposure beginning in late childhood, suggesting that digital habits are being established before regulatory capacities are fully consolidated. The normalization of smartphone access among school-going adolescents, including those in government and government-aided institutions, reflects broader socio-technological shifts within the Indian context. Smartphone use is no longer restricted to higher socioeconomic strata but has become structurally embedded in educational and social functioning.

Nearly half of the sample resided in single-parent households, enabling examination of relational variables across diverse family structures. This distribution strengthens the ecological validity of the findings and allows attachment-related processes to be interpreted across varying caregiving contexts.

Importantly, adolescents reported mixed-purpose use—education, entertainment, and social communication, indicating functional integration rather than purely recreational engagement. However, functional utility does not preclude dysregulation. As documented in global research, multifunctional devices increase exposure frequency, habitual checking behaviors, and reinforcement density (15, 36). Taken together, these patterns position problematic smartphone use as a systemic developmental concern rather than a discrete behavioral anomaly, warranting public health framing and preventive intervention.

### 4.2 Demographic influences: exposure and gender matter

Consistent with prior literature, male adolescents exhibited significantly higher levels of smartphone addiction severity. Logistic regression analyses further indicated that males were approximately three times more likely to fall within the at-risk category. This magnitude of effect suggests not merely a mean-level difference but a clinically meaningful disparity in vulnerability.

One explanatory pathway lies in differential patterns of engagement. Male adolescents are more likely to engage in reward-intensive digital activities such as competitive gaming and performance-oriented applications, which operate on variable reinforcement schedules and social ranking systems (26). Such environments amplify dopaminergic reward sensitivity, a system already heightened during adolescence, thereby increasing susceptibility to compulsive engagement.

In addition to gender, exposure duration emerged as a significant predictor. Each additional year of smartphone use increased addiction risk, indicating a cumulative reinforcement process. Early and sustained exposure may consolidate habitual responding through repeated cue–reward associations, while simultaneously reducing opportunities to strengthen offline regulatory competencies. From a self-regulation framework, prolonged digital immersion may interfere with the development of delay tolerance and effortful control.

These findings highlight two key implications. First, risk is not solely a function of current usage intensity but also of developmental timing and exposure duration. Second, preventive efforts must begin earlier than typically assumed. Digital literacy programs should address not only screen-time limits but also reward awareness, impulse regulation, and balanced engagement strategies during late childhood and early adolescence.

Collectively, the results suggest that demographic influences operate through both structural (gender-linked engagement patterns) and developmental (exposure duration) pathways, shaping vulnerability to problematic smartphone use.

### 4.3 Maternal support as a protective relational factor

In the maternal model, perceived maternal support emerged as a modest but significant protective factor. Adolescents who experienced greater trust and communication with their mothers reported lower smartphone addiction severity, even after controlling for demographic characteristics.

This finding aligns with attachment-based perspectives emphasizing the regulatory function of emotionally responsive caregiving. When adolescents experience relational security, reliance on digital environments for emotional compensation may be reduced. Notably, maternal alienation did not independently predict addiction in the adjusted model. This suggests that supportive maternal engagement may be more influential than the absence of conflict. It also aligns with the conceptualization of parental support as reflecting emotional availability, a construct theoretically grounded in attachment research. Combining trust and communication into a composite support variable is therefore consistent with established attachment frameworks, where these dimensions jointly represent relational security rather than discrete processes.

### 4.4 Paternal alienation: a distinct relational vulnerability

The most consistent relational finding was the role of paternal alienation. Higher perceived alienation from fathers significantly predicted smartphone addiction severity beyond age, gender, exposure duration, and family structure.

Notably, paternal support was not significant in the adjusted model, whereas paternal alienation was. This asymmetry suggests that negative relational experiences may be more strongly associated with problematic digital engagement than positive ones within the paternal context. Perceived emotional withdrawal may heighten distress or dysregulation, increasing susceptibility to reward-driven or avoidance-based smartphone use.

The present findings may be particularly relevant within the Indian sociocultural context, where paternal roles are often characterized by authority, instrumental provision, and comparatively lower emotional expressiveness. In rapidly urbanizing regions such as Chennai, adolescents experience increasing digital autonomy alongside traditional family structures. Within such contexts, perceived paternal emotional distance may carry amplified psychological significance, potentially increasing adolescents’ reliance on digital environments for social validation or emotional regulation. These findings, therefore, contribute contextually grounded evidence from an underrepresented population in behavioral addiction research.

### 4.5 Family structure: contextual but not an independent predictor

Family structure did not independently predict smartphone addiction severity in the final regression models. Although a small association emerged in the preliminary maternal model (Model 1), this effect was no longer significant after relational variables were included. This pattern indicates that relational quality accounts for the variance initially attributed to structural configuration.

These findings align with research suggesting that emotional processes within families are more strongly associated with adolescent adjustment than structural arrangements alone. Living in a single- or dual-parent household, in itself, does not appear to determine vulnerability to problematic smartphone use once relational factors are considered.

Although family structure represents an important contextual characteristic of adolescents’ living arrangements, no statistical evidence of a moderating or independent effect was observed in the present analyses. The findings, therefore, emphasize relational dynamics, rather than structural configuration, as the more proximal correlate of smartphone addiction severity.

### 4.6 Continuous severity versus categorical risk

An important nuance in the findings is that paternal alienation predicted continuous addiction severity but did not reach statistical significance in predicting categorical risk status. This distinction suggests that relational factors may influence the degree of problematic engagement rather than necessarily determining whether an adolescent crosses a diagnostic threshold.

This reinforces the value of dimensional approaches in behavioral addiction research, where relational stressors may contribute to escalating severity even if they do not independently classify individuals into risk categories.

### 4.7 Integrating findings within theoretical frameworks

The findings can be interpreted through complementary theoretical frameworks. Attachment theory posits that secure parent–child relationships support emotional stability and internal regulation (17, 18). When adolescents perceive relational disconnection, particularly paternal alienation, regulatory resources may be compromised.

Compensatory models of digital engagement suggest that individuals may increase online involvement to manage unmet psychological needs or negative affect (19). Within this framework, paternal alienation may function as a relational stressor associated with greater reliance on smartphones for short-term emotional regulation.

Although the additional variance explained by relational variables was modest (*ΔR^2^* ≈ 0.03–0.06), this pattern is consistent with multifactorial models of behavioral outcomes, where many small contextual influences accumulate. Together with demographic and exposure-related factors, relational quality appears to be one such contributing layer rather than a dominant determinant.

## 5 Public health implications

This study highlights several priorities for adolescent mental health and digital well-being initiatives:

- Relationally informed prevention: Interventions may benefit from integrating parent–adolescent communication enhancement and emotionally responsive father involvement, emphasizing relational quality rather than structural family configuration.
- Early digital literacy education: Longer smartphone use duration increased the risk of addiction. Schools may consider implementing structured digital health literacy programs that promote self-regulation, balanced use, and awareness of problematic patterns.
- Gender-sensitive approaches: Male adolescents showed higher addiction risk. Prevention efforts may benefit from addressing motivational and behavioral patterns associated with digital engagement while avoiding stereotypes.
- Community-level support systems: Accessible family-based psychoeducation initiatives and community mental health resources may help strengthen relational dynamics that buffer against digital overuse.

## 6 Limitations

Several limitations should be considered when interpreting these findings:

- Cross-sectional design: causal direction cannot be established. Relational strain may contribute to smartphone addiction, but excessive smartphone use may also affect family dynamics.
- Self-report measures: all variables were based on adolescent perceptions, which may introduce shared method variance.
- Regional sampling: participants were drawn from government and government-aided schools within a single metropolitan region, limiting generalizability to private schools, rural areas, or other Indian states.
- Family structure restriction: sample limited to adolescents living with ≥1 biological parent, excluding grandparent/guardian caregiving arrangements common in India.
- Unmeasured variables: factors such as peer attachment, parental monitoring practices, emotion regulation, and parental digital behavior were not assessed and may influence outcomes.

Accordingly, findings should be interpreted within the methodological constraints of cross-sectional and self-reported data. Despite these limitations, hierarchical modeling clarified the relative contributions of demographic and relational variables.

## 7 Recommendations for future research and intervention

- Longitudinal tracking: future research should employ longitudinal designs to clarify the developmental directionality between parental relationship quality and smartphone addiction severity.
- Expanding relational dynamics: multi-informant studies incorporating parent, peer, and teacher perspectives may provide a more comprehensive understanding of relational processes. Examining parental smartphone use and “technoference” as potential mediating or moderating mechanisms would further clarify family dynamics in the digital realm.
- Relationally focused family interventions: prevention programs may benefit from strengthening parent–adolescent communication, particularly promoting emotionally responsive father involvement. Future interventions could evaluate whether improvements in relational quality reduce problematic smartphone engagement over time.
- School-based preventive supports: integrating digital literacy, emotion regulation training, and healthy coping strategies into school curricula may help adolescents develop balanced patterns of digital engagement.

## 8 Conclusion

This study examined adolescent smartphone addiction within the broader emotional context of parent–adolescent relationships. While male gender and longer duration of smartphone use emerged as consistent predictors of addiction severity and risk, relational quality also played a meaningful role. Maternal support demonstrated a modest protective association, whereas perceived paternal alienation was linked to greater addiction severity beyond demographic factors. Importantly, family structure did not independently predict outcomes once relational variables were considered, indicating that emotional relationship quality was more strongly associated with addiction severity than structural configuration alone. These findings suggest that understanding adolescent smartphone addiction requires attention not only to behavioral exposure but also to the emotional environment in which digital engagement occurs. Strengthening parent–adolescent connection may therefore represent a valuable complement to traditional screen-time regulation strategies in promoting adolescent well-being.

## Data Availability

The raw data supporting the conclusions of this article will be made available by the authors, without undue reservation.
